# Cyromazine affects the ovarian germ cells of *Drosophila via* the ecdysone signaling pathway

**DOI:** 10.3389/fphys.2022.992306

**Published:** 2022-09-29

**Authors:** Muhammad Zaryab Khalid, Zhipeng Sun, Jing Zhang, Shijie Zhang, Guohua Zhong

**Affiliations:** Key Laboratory of Natural Pesticide and Chemical Biology, Ministry of Education, South China Agricultural University, Guangzhou, China

**Keywords:** germline stem cells, primordial stem cells, RNA sequencing, RNA interference, ecdysone signaling, apoptosis

## Abstract

Cyromazine, an insect growth regulator, has been extensively used against the insect pests of livestock and households. Previously, it was observed that the continuous selection of cyromazine from the larval to the adult stage decreased the number of germline stem cells (GSCs) and cystoblasts (CBs) in the adult ovary. In addition, in this study, we observed that the number of primordial germ cells (PGCs) was also decreased in the larval ovary after treatment with cyromazine. However, the mechanism by which it affects the germ cells is yet to be explored. Consequently, to deeply investigate the effects of cyromazine on the germ cells, we performed tissue-specific RNA sequencing. Bioinformatics analysis revealed that the ecdysone signaling pathway was significantly influenced under cyromazine stress. Based on that, we screened and selected 14 ecdysone signaling responsive genes and silenced their expression in the germ cells only. Results of that showed a considerable reduction in the number of germ cells. Furthermore, we mixed exogenous 20E with the cyromazine-containing diet to rescue the ecdysone signaling. Our results supported that the application of exogenous 20E significantly rescued the germ cells in the transgenic lines. Therefore, this implies that the cyromazine decreased the number of germ cells by affecting the ecdysone signaling pathway.

## 1 Introduction

**FIGURE 1:**
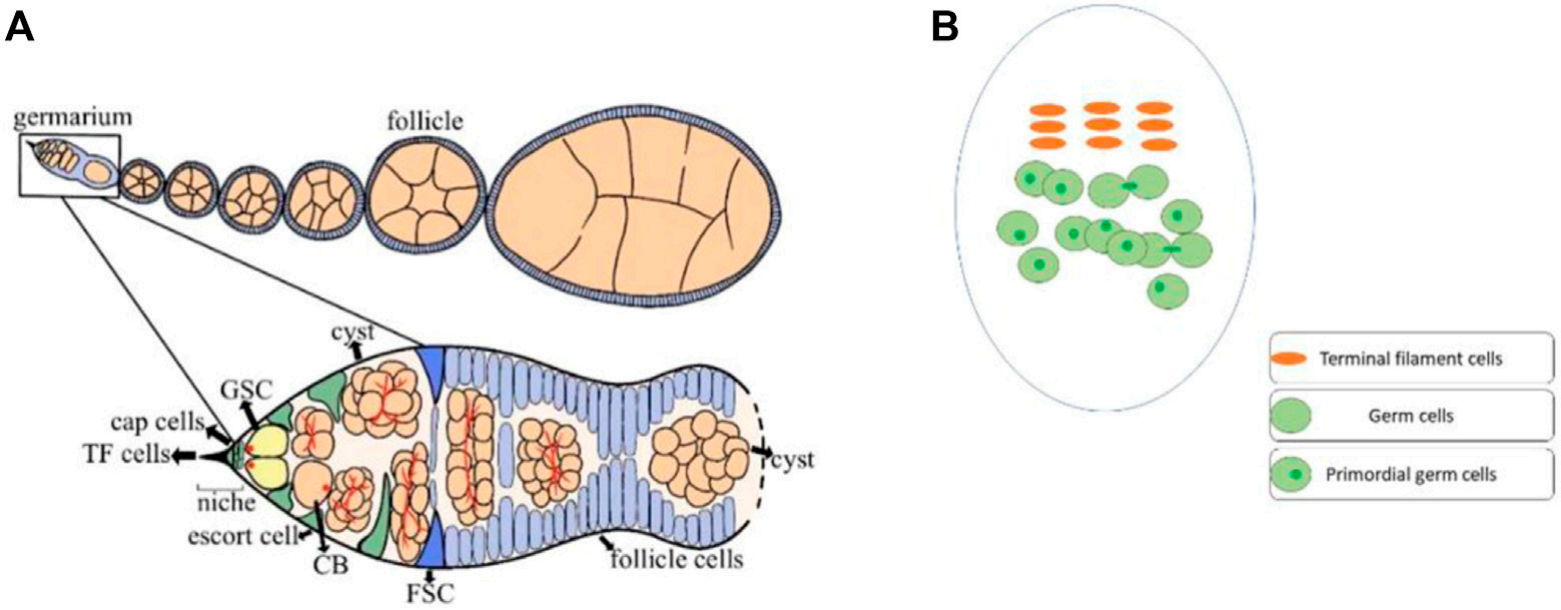
Ovaries of *Drosophila* house different types of cells. **(A)** The adult ovary of *Drosophila* consists of 16–20 ovarioles, and the germarium of each ovariole has different types of cells. The germline stem cells (GSCs) strictly maintain and differentiate under the regulation of signals from the somatic cells. At every stage of early cell division, the GSCs are surrounded by somatic cells for their proper regulation. GSCs divide into cystoblasts (CBs), which later undergo four rounds of mitotic division to generate a 16-cells cyst. In the end, only one cell among these 16-cell cysts differentiates as an oocyte. **(B)** The third instar larval ovary of *Drosophila*, also consists of somatic cells and germ cells. Before pupation, most of the terminal filaments (TF) cells has formed. While the TF cells completely formed at the early pupal stage. The germ cells can be identified with a germ cell-specific marker (anti-vasa). However, only the germ cells with single dot-shaped spectrosome were considered as PGCs.

Reproduction is an inherent ability of organisms by which they multiply. *Drosophila melanogaster*’s ovary is an excellent model system for studying the germ cell’s proliferation and differentiation (Ting, 2013). The primordial germ cells (PGCs) continue to increase during the larval ovary development (Gilboa and Lehmann, 2006), while the terminal filament (TF) cells are completely formed at the early pupal stage. However, very few cap cells had formed at this early pupal stage (Zhu and Xie, 2003). It is worth mentioning that both TF and cap cells play a crucial role in the maintenance of PGCs by producing Decapentaplegic (Dpp) and Hedgehog (Hh) signaling (member of bone morphogenetic protein) (Sato et al., 2010). In adults of *Drosophila*, the ovary consists of 16–20 ovarioles and 2–3 germline stem cells (GSCs) are found in the germarium. These germ cells are regulated by a number of GSCs specific intrinsic factors, and extrinsic niche signals (Ji et al., 2017; Weaver and Drummond-Barbosa, 2018; Gao et al., 2019; Yoshinari et al., 2020). Furthermore, the GSC niche is formed of different types of somatic cells, including TF, cap cells, and escort cells (ECs) (Eliazer and Buszczak, 2011; Ben-Zvi and Volk, 2019; Drummond-Barbosa, 2019). These niche cells also produce Dpp and Hh signaling, which is necessary for the maintenance and differentiation of GSCs (Liu et al., 2015). In the germarium, GSCs are connected with cap cells through E-cadherin, the loss of which results in a sharp decline in the GSC number (Jin et al., 2008). Each niche signaling molecule is crucial for the maintenance and differentiation of GSCs (Huang et al., 2017; Panchal et al., 2017; Mao et al., 2019). For example, loss of *Traffic jam* (*Tj*), a Maf transcriptional factor, from cap cells not only results in the loss of cap cells but also causes a reduction in GSCs. However, *Tj* loss from escort cells arrests cystoblast (CB) differentiation (Li et al., 2019). Thus, under the strict regulation of these signaling factors, the GSCs divide asymmetrically into CBs, which later complete four rounds of mitotic divisions to produce 16-cell cysts (Hinnant et al., 2020). While the early dividing gem cells are accompanied by escort cells, the newly developed 16-cell cyst, on the other hand, is enveloped by follicle cells (Figure 1) (Morris and Spradling, 2012; Eliazer et al., 2014).

Cyromazine is an insect growth regulator, which is among the most widely used biorational insecticides (Khan and Akram, 2017). It is used to control the important pests of livestock, including *Stomoxys calcitrans* and *Lucilia cuprina*, and also to control *Musca domestica*, by affecting the immature stage of the fly (Taylor et al., 2012). The treatment of *D. melanogaster*’s larvae with cyromazine resulted in the early emergence of adults. Furthermore, the mode of action of cyromazine is thought to be related to 20-hydroxyecdysone (20E), as the exogenous application of 20E decreased larval mortality (Van De Wouw et al., 2006). Furthermore, the number of GSCs was significantly reduced in the *EcR* mutant females, indicating that the ecdysone signaling directly controls the GSC maintenance and proliferation (Ables and Drummond-Barbosa, 2010).

Our previous study showed that the continuous selection of *D. melanogaster* from the larval to the adult stage affected the GSCs and CBs in the adult ovary. Furthermore, we observed that the expression of selected ecdysone signaling-related genes and ecdysone titer significantly decreased in the treated ovaries (Khalid et al., 2022). Therefore, to deeply investigate how this chemical affected the germ cells in the adult ovaries, we first counted the number of PGCs in the larval ovaries. Our results indicated a significant decrease in the number of PGCs compared to the control group. Thus, we concluded that the continuous selection of cyromazine resulted in a significant decrease in the number of germ cells from the larval to the adult stage. Later, we performed tissue-specific next-generation RNA sequencing of both larval and adult ovaries, to screen genes involved in the ecdysone signaling pathway under cyromazine stress. Furthermore, based on our RNA-Seq result, we selected 14 ecdysone signaling responsive genes and performed germ cell-specific RNA interference (RNAi) of selected genes to functionally study the effect of cyromazine on the germ cells of *D. melanogaster*. In addition, the exogenous application of 20E significantly rescued the germ cells in both larval and adult ovaries, which further confirmed that the effect of cyromazine, on the germ cells of *Drosophila*, is through the ecdysone signaling pathway. We further performed the TUNEL assay to find out if the decrease in the number of germ cells is due to cell death. However, no positive apoptotic signal was observed as compared to the control group, indicating that the decrease in the number of germ cells is not due to cell death.

## 2 Materials and methods

### 2.1 *Drosophila* strains and breeding conditions

Standard cornmeal agar medium was used to rear the flies at a constant temperature of 25°C and humidity of 75% with a 12:12 h light/dark cycle. The details of the transgenic lines used in the study are provided (Supplementary Table S1).

### 2.2 Insecticide treatment and next-generation RNA sequencing

Technical-grade cyromazine was purchased from Guangzhou Qixiang Biotechnology Co., Ltd. (Guangzhou). The stock solution of 1 mg/ml cyromazine dissolved in distilled water was used for serial dilutions. The required volume of cyromazine was mixed with a freshly prepared diet before solidification. The larvae of *D. melanogaster* were allowed to feed on 0.3 PPM of cyromazine for 12 h, while after the adult emergence, the adults were fed on a diet containing 50 PPM.

At the late third instar larvae, we carefully dissected ovaries from the control and insecticide-treated groups. The adult ovaries were dissected after 7 days of adult emergence. All the ovaries were dissected in phosphate buffer saline (PBS) with a sterilized dissection kit and immediately frozen in liquid nitrogen. The Trizol Reagent Kit (Invitrogen, Carlsbad, CA, United States) was used to extract total RNA following the manufacturer’s protocol. The integrity of RNA was measured on Agilent 2100 Bioanalyzer (Agilent, Palo Alto, CA, United States). mRNA was enriched by magnetic beads with Oligo (dT). Later, mRNA was fragmented and reverse-transcribed into cDNA by fragmentation buffer and random primers, respectively. While for synthesizing the second-strand cDNA, RNase H, dNTP, DNA polymerase I, and buffer were used. The QiaQuick PCR Extraction Kit (Qiagen, Venlo, Netherlands) was used to purify the cDNA fragments, end-repaired, poly (A) added, and ligated to Illumina sequencing adapters. Agarose gel electrophoresis was used to recover the target fragment, PCR amplified, and sequenced using Illumina HiSeq 2500 by gene Denovo Biotechnology Co. (Guangzhou, China).

### 2.3 Bioinformatics analysis

High-quality clean reads were acquired through removing adapters above 10% of unknown nucleotides (N) and low-quality reads with more than 50% of low-quality (Q-value ≤ 20) bases using fastp (version 0.18.0). Later, Bowtie2 (version 2.2.8) was used, and reads were mapped to the ribosome RNA (rRNA) database. Mapped reads were then removed, and clean reads were mapped to *D. melanogaster*’s reference genome (GCF_000001215.4) using HISAT2 (Kim et al., 2015). DESeq2 software was used for differential expression analysis, while a false discovery rate (FDR) below 0.05 and absolute fold change ≥2 were used to identify differentially expressed gene/transcripts. Furthermore, for functional annotation, both Gene Ontology (GO) enrichment analysis and Kyoto Encyclopedia of Gene and Genomes (KEGG) pathway enrichment analysis of all predicted genes were performed. However, FDR correction was followed for the *p*-value, where FDR ≤ 0.05 was taken as a threshold. However, GO terms and KEGG pathways with corrected *p*-value < 0.05 were considered as significantly enriched among differentially expressed genes (DEGs) (Cao and Jiang, 2017).

### 2.4 Validation through RT-qPCR

For validating the RNA-Seq results, real-time quantitative PCR (RT-qPCR) was performed on 14 DEGs that were differentially expressed in larval and adult ovaries. Total RNA was extracted from both larval and adult ovaries, and cDNA was synthesized by using the PrimeScript RT reagent Kit containing gDNA eraser (Takara, China). Later, RT-qPCR was performed by using SsoFast EvaGreen Supermix (Bio-Rad, Hercules, CA, United States). The following working program was set as 2 min at 95°C, 40 cycles of 5 s at 95°C, 10 s at 60°C, and a melting curve from 65 to 95°C (Zafar et al., 2021). For internal control, several housekeeping genes were investigated for normalization according to the 2^−ΔΔ^CT method, and finally, *rp49* was selected. Primers were constructed by Primer Premier 5 (software) and are provided (Supplementary Table S2).

### 2.5 Germ cell-specific RNAi-mediated gene silencing

From our RNA-sequencing results and based on the previously published articles, we selected 14 ecdysone-responsive genes for RNAi-mediated gene silencing experiments. Furthermore, we divided these lines into three categories (primary genes, Halloween genes, and ecdysone-responsive genes). Later, the virgin females of nos-Gal4 were crossed with the males of *UAS-RNAi* transgenic flies to drive the RNAi-mediated gene silencing in the germ cells. While for control, the virgin females of nos-Gal4 were crossed with the males of yw. In total, 10 males together with 10 females were kept in each vial, and all the vials were maintained at 25°C. Both the larvae and adults were allowed to feed on the cyromazine mixed diet. The late 3rd instar larvae were used to dissect larval ovaries, while adult ovaries were dissected after 7 days of eclosion.

### 2.6 Immunohistochemistry and microscopy

Antibody staining was performed as previously described (Liu et al., 2015). In brief, the ovaries were first fixed in 4% paraformaldehyde (PFA), washed three times with PBST (0.1% Triton X-100 in PBS), blocked in 5% normal goat serum (NGS), and incubated with primary antibodies at 4°C overnight. The next day, ovaries were washed with PBST three times, blocked in 5% NGS, incubated with secondary antibodies for 2 h, and washed with PBST three times. In addition, Hoechst (1:5,000; Cell Signaling Technology, Danvers, MA, United States) was used to stain the DNA. The samples were then mounted in 90% glycerol (Sigma).

Following primary antibodies were used: rabbit anti-pMad (1:400; cell signaling), mouse anti-α-Spectrin [3A9, 1:100; Developmental Studies Hybridoma Band (DSHB)], and rabbit anti-vasa (1:3,000). Secondary antibodies used were rabbit cy3 (1:1,000) and mouse 488 (1:1,000). Nikon A1 plus confocal microscope was used to take images (Nikon, Tokyo, Japan).

### 2.7 Counting the number of germ cells

At the larval stage, the germ cells are known as PGCs, which are identified on the base of germ cell exclusive proteins such as vasa (Yatsenko and Shcherbata, 2021). Therefore, for counting the PGCs, we stained the larval ovaries with both anti-vasa and anti-α-Spectrin (3A9). The germ cells having a single dot-shaped spectrosome were counted as PGCs. While for counting the number of GSCs in the adults, we used anti-pMad. The expression of pMad is used as a marker for GSCs (Liu et al., 2015). So, we stained the adult ovaries with both anti-pMad and anti-α-Spectrin (3A9). Only the GSCs and CBs express a single round spectrosome. So, the CBs were identified from the dividing cells based on the presence of a single dot containing spectrosome.

### 2.8 Rescue experiment by feeding exogenous 20E

20E was purchased as a stock solution of 10 mM/ml (CAS 5289-74-7, Shanghai Taoshu Biotechnology Co., Ltd.). For the rescue experiment, to further validate the effect of cyromazine on the germ cells, exogenous 20E was mixed with the food containing cyromazine at a final concentration of 500 μM. The 20E was uniformly mixed with the food by continuously blending it for 3 min. The number of germ cells was then counted from both the larval and adult ovaries.

### 2.9 TUNEL assay to detect cell death

To detect whether the feeding of cyromazine caused the cell death of the germ cells, we used the *in situ* cell death detection kit (Roche, Mannheim, Germany, 11684795910). Concisely, the ovaries were fixed in 4% paraformaldehyde for 20 min, followed by washing for 30 min and incubation with TUNEL reaction mixture (containing label solution and enzyme solution) for 60 min at 37°C. Later, blocking was performed in NGS and immunofluorescence staining was followed as described in Section 2.6.

### 2.10 Statistical analysis

Excel (Microsoft) and Prism 9.0 (GraphPad) were used to record the statistical data. Student’s *t*-test was used to determine *p*-values, and *p*-values are provided in comparison with the control except for the rescue experiment. For the *t*-test, *p < 0.05; **p < 0.01; ***p < 0.001.

## 3 Results

### 3.1 RNA-sequencing analysis

We used next-generation Illumina RNA sequencing for 12 libraries, including insecticide-treated and control groups, which generated 164,933,474 (CK-L), 172,672,572 (T-L), 140,249,820 (CK-A), and 166,956,380 (T-A) raw quality reads. Later, 164,335,506 (CK-L), 172,167,904 (T-L), 139,912,686 (CK-A), and 166,568,432 (T-A) of high-quality clean reads were obtained by data filtering through fastp. The percentage clean reads ratio of all libraries was higher than 99%, with an average of 99.71%. The Q20 value of clean base pairs of all 12 libraries was higher than 97.30%, displaying good quality clean reads. The Q30 value and GC content of each replication are also shared (Supplementary Table S3). Clean reads were then mapped to the *D. melanogaster* genome (GCF_000001215.4), and the percentage of mapped reads ranged from 95.49% to 97.01%.

### 3.2 Identification of differentially expressed genes

The FPKM value was used to identify DEGs between the control and the insecticide-treated groups. A total of 863 genes were considered as DEGs between the control and insecticide-treated adult ovaries, among which 85 genes were upregulated and 778 genes were downregulated (Supplementary Table S4). However, a total of 26 genes were considered as DEGs among control and treated larval ovaries, where 24 genes were upregulated and only two genes were downregulated (Supplementary Table S5). In addition, we used the GO enrichment analysis to understand the functions of our target genes in cellular components, biological processes, and molecular functions (Supplementary Figures S1, S2), while the KEGG pathway enrichment analysis was used to cluster genes into different pathways (Supplementary Figures S3, S4). The grouped genes of the same pathway normally participate in the same biological process.

The ecdysone signaling controls the germ cell maintenance and differentiation (Ables and Drummond-Barbosa, 2010). Therefore, in this study, we selected 14 key genes from the ecdysone signaling pathway to validate the effect of cyromazine on the germ cells of *Drosophila*’s ovary.

### 3.3 RT-qPCR validation of RNA-seq

We randomly selected DEGs from both larval and adult transcriptomic data to validate RNA-Seq results by RT-qPCR. From larval transcriptomic data, we selected *Lcp4*, *TotC*, *onecut*, *lectin-37Da*, *LysS*, *Hsp70Aa*, and *Lcp65Ab1*, while from adult transcriptomic data, we randomly selected *Iva*, *blw*, *mus312*, *NLK*, *Act88F*, *Diedel*, and *DptA*. The mRNA expression level is presented in Supplementary Figure S5. Overall, the results showed reliability between the RNA-Seq and RT-qPCR.

### 3.4 Cyromazine affects the germ cells in the larval ovary

During larval development, female PGCs progress to proliferate instead of differentiating (Sato et al., 2010). An important differentiation factor, *Bag of Marbles (Bam)*, is not expressed before the early pupal stage (Gilboa and Lehmann, 2004). To identify the PGCs, we stained germ cells with mouse anti-α-Spectrin (3A9) and rabbit anti-vasa antibodies (Gilboa and Lehmann, 2006; Sato et al., 2010; Gancz et al., 2011). Later, we carefully counted the number of PGCs. However, germ cells with a single dot containing spectrosome were counted as PGCs (Yatsenko and Shcherbata, 2021).

**FIGURE 2:**
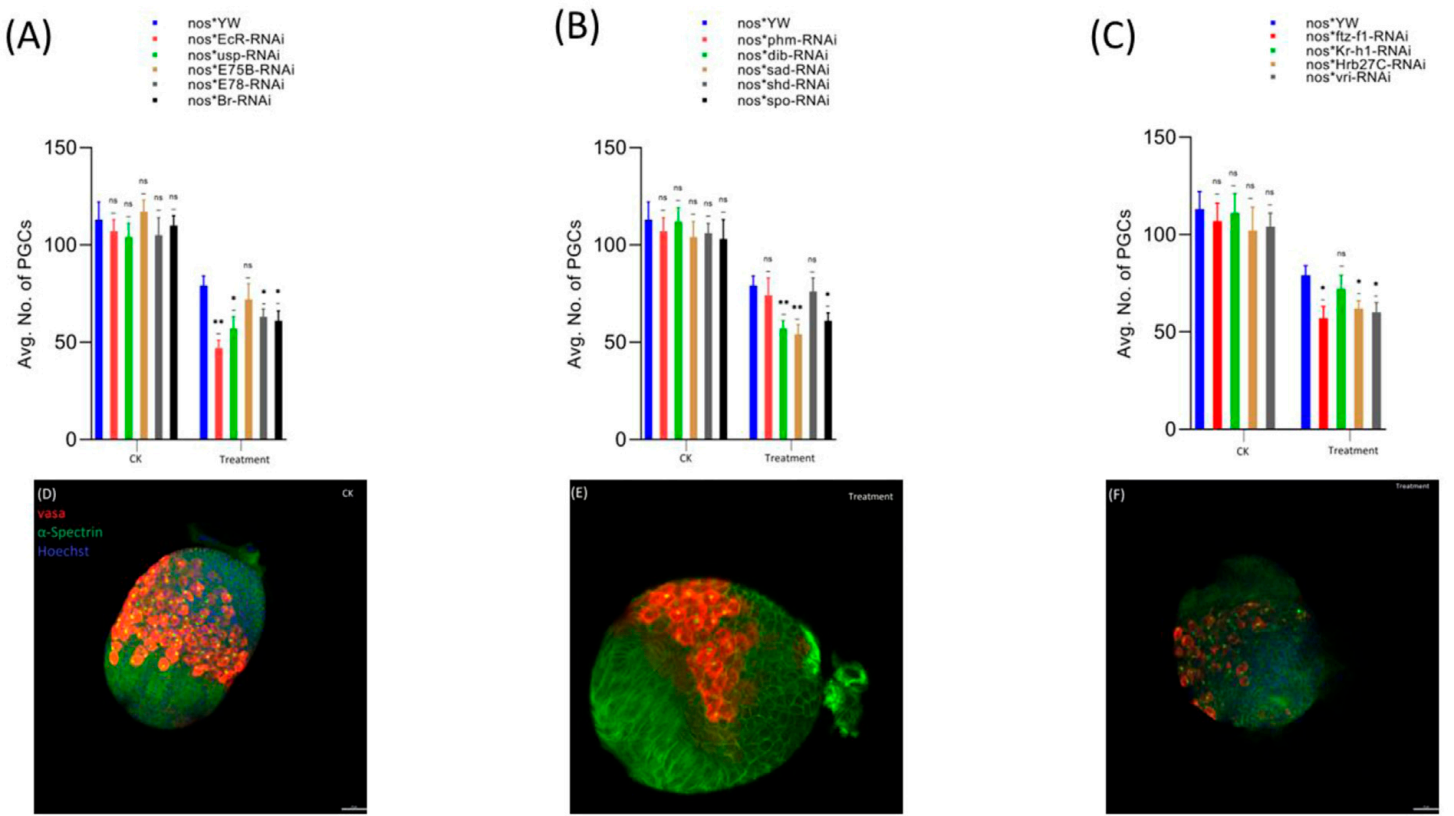
Effect of cyromazine on the germ cells of larval ovaries. **(A)** RNAi of ecdysone-responsive primary genes and the effect of cyromazine on the PGCs. **(B)** RNAi of Halloween genes and the effect of cyromazine on the PGCs. **(C)** RNAi of ecdysone-responsive genes and the effect of cyromazine on the PGCs. CK means that the flies were fed on a normal diet, while treatment means the flies were fed insecticide containing diet. **(D)** Larval ovary from the CK group. **(E,F)** Larval ovaries from the cyromazine-treated group (scale bar, 25 μm). For the *t*-test: *p < 0.05; **p < 0.01; ***p < 0.001; ns indicates not significant (*p* > 0.05).

First, we observed that the treatment with cyromazine significantly decreased the number of PGCs in the insecticide-treated ovaries (79 ± 5) of nos-Gal4 * yw, as compared to the control (113 ± 9). Later, to further investigate if cyromazine affects the germ cells of *D. melanogaster* by the ecdysone signaling pathway, we performed germ cell-specific RNAi of selected ecdysone signaling responsive genes by using germ cell-specific driver. Results indicated that knockdown of selected genes significantly decreased the number of PGCs in the cyromazine-treated ovaries as compared to the control group (Figure 2).

Overall, knockdown of these ecdysone-responsive genes further decreased the number of PGCs as compared to the nos-Gal4 * yw in the cyromazine-treated group, implying that the effect of cyromazine on the PGCs is through the ecdysone signaling pathway. Furthermore, the maximum numbers of PGCs were decreased against *EcR* mutant flies (Figure 2A) followed by *sad* and *dib* mutant flies (Figure 2B), while the least numbers of PGCs were decreased against *shd* mutant flies (Figure 2B).

### 3.5 Cyromazine affects the germline stem cells and cystoblasts in the adult ovary

**FIGURE 3:**
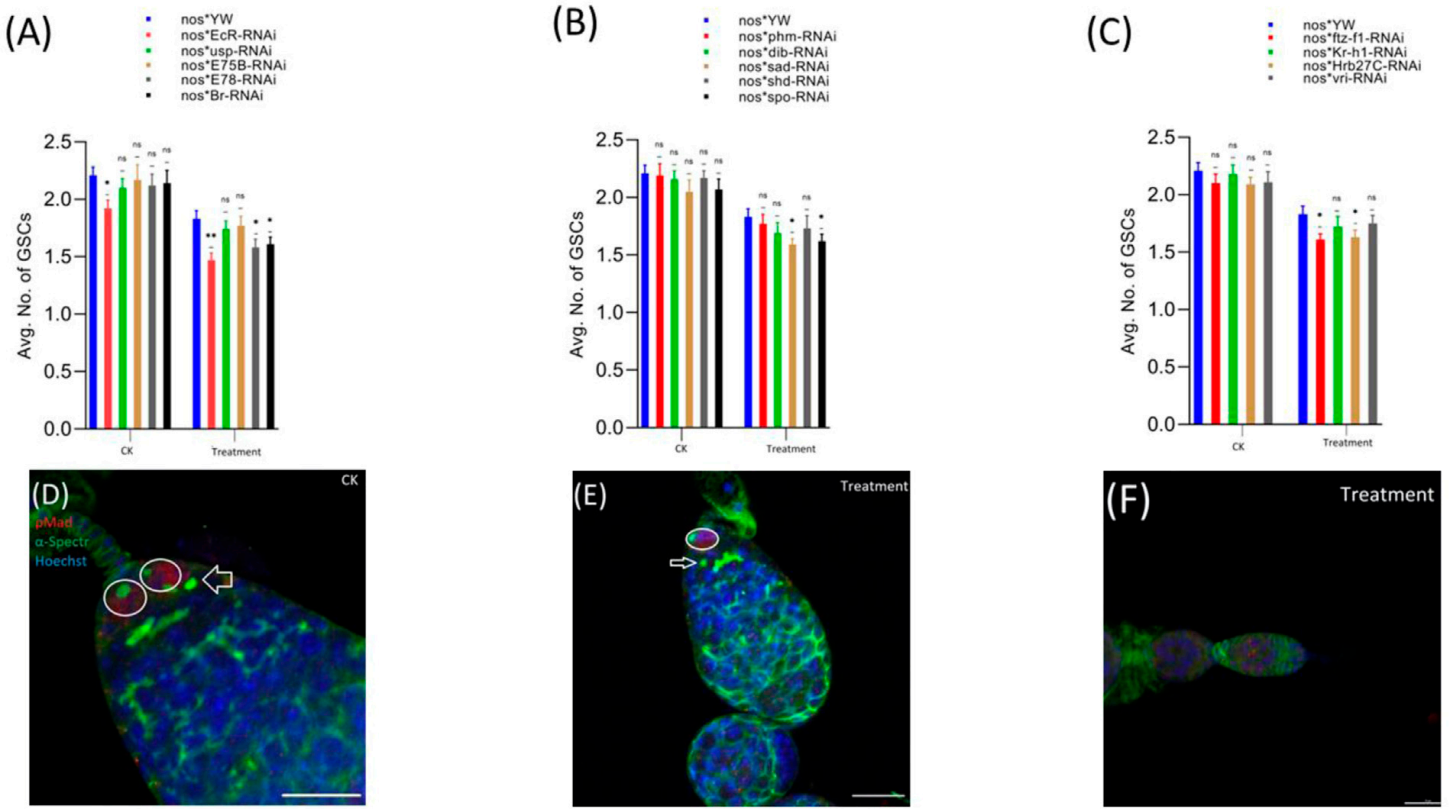
Effect of cyromazine on the GSCs of adult ovaries. **(A)** RNAi of ecdysone-responsive primary genes and the effect of cyromazine on the GSCs. **(B)** RNAi of Halloween genes and the effect of cyromazine on the GSCs. **(C)** RNAi of ecdysone-responsive genes and the effect of cyromazine on the GSCs. CK means that the flies were fed on a normal diet, while treatment means the flies were fed insecticide containing diet. **(D)** The adult ovariole from the CK group. White circle indicates GSCs, while white arrow represents CBs. **(E)** Only one GSCs and one CB is present in the cyromazine-treated ovariole. **(F)** The ovariole lacking any GSC in the germarium (scale bar, 10 μm **(D,E)**, 25 μm **(F)**). For the *t*-test: *p < 0.05; **p < 0.01; ***p < 0.001; ns indicates not significant (*p* > 0.05).

**FIGURE 4:**
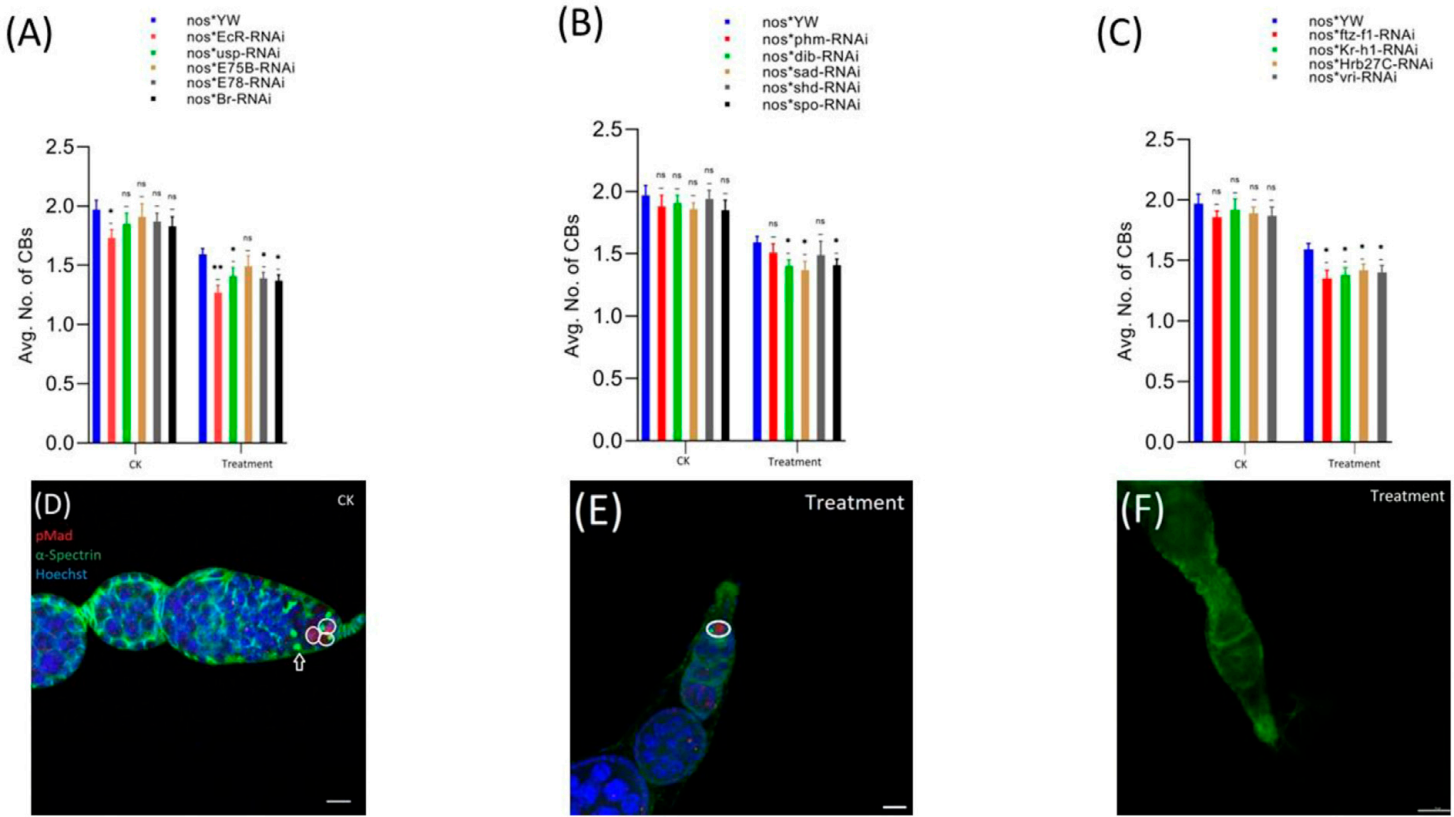
Effect of cyromazine on the CBs of adult ovaries. **(A)** RNAi of ecdysone-responsive primary genes and the effect of cyromazine on the CBs. **(B)** RNAi of Halloween genes and the effect of cyromazine on the CBs. **(C)** RNAi of ecdysone-responsive genes and the effect of cyromazine on the CBs. CK means that the flies were fed on a normal diet, while treatment means the flies were fed insecticide containing diet. **(D)** The adult ovariole from the CK group. White circle indicates GSCs, while white arrow represents CBs. **(E)** Only one GSCs and no CB can be seen against the cyromazine-treated ovariole. **(F)** The ovariole lacking any germ cell in the germarium (scale bar, 10 μm **(D,E)**, 25 μm **(F)**). For the *t*-test: *p < 0.05; **p < 0.01; ***p < 0.001; ns indicates not significant (*p* > 0.05).

pMad expression is highly specific for the GSCs. Therefore, we stained the GSCs with anti-pMad and anti-α-Spectrin (3A9). The CBs were identified based on the presence of a single spherical spectrosome (Figure 3D). We observed that cyromazine significantly reduced both the GSCs and CBs in the female adult ovary of nos-Gal4 * yw, as compared to the control (Figures 3, 4).

In addition, RNAi results showed that knockdown of the selected genes significantly reduced the numbers of both GSCs and CBs in the cyromazine-treated group, as compared to the control group. While knockdown of ecdysone-responsive genes among the cyromazine-treated group further decreased the number of both GSCs and CBs as compared to the nos-Gal4 * yw, implying that the effect of cyromazine on the germ cells is through the ecdysone signaling pathway. Maximum number of GSCs were decreased against *EcR* mutant flies (Figure 3A) followed by *E78* and *sad* mutant flies (Figures 3A,B), among the cyromazine-treated group, while least numbers of GSCs decreased in *phm* mutant flies (Figure 3B). Furthermore, cyromazine significantly decreased the number of GSCs against the ecdysone-responsive genes (Figure 3C). Many germerium contain only one GSC (Figure 3E), while few germarium lack any germ cell (Figure 3F).

Likewise, *EcR* mutant flies (Figure 4A) displayed the highest decrease in the number of CBs followed by *ftz-f1* and *Br* mutant flies (Figures 4A,C), while *phm* mutant flies displayed the minimum decrease in the number of CBs, among the cyromazine-treated group (Figure 4B).

### 3.6 Rescue experiment

**FIGURE 5:**
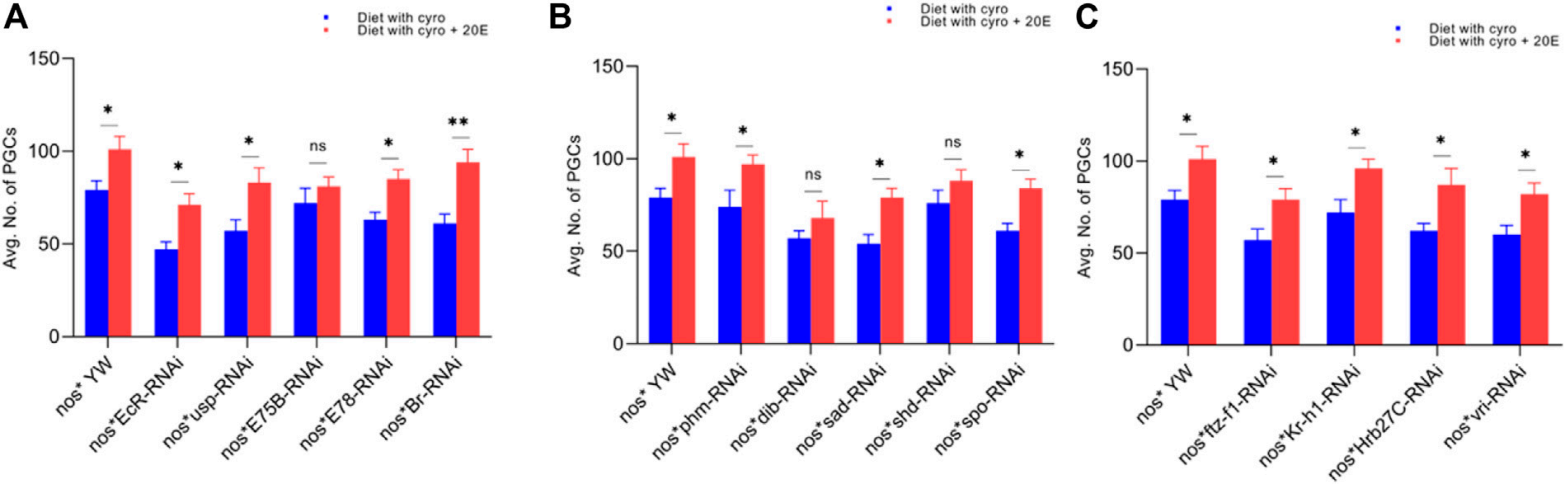
Rescue experiment of PGCs. **(A)** The number of PGCs against ecdysone-responsive primary genes. **(B)** The number of PGCs against Halloween genes. **(C)** The numbers PGCs against ecdysone-responsive genes. The blue bars indicate the numbers of PGCs against the cyromazine treatment, while the red bars indicate the numbers of PGCs from the flies fed on a diet containing 20E with the cyromazine. For the *t*-test: *p < 0.05; **p < 0.01; ***p < 0.001; ns indicates not significant (*p* > 0.05).

**FIGURE 6:**
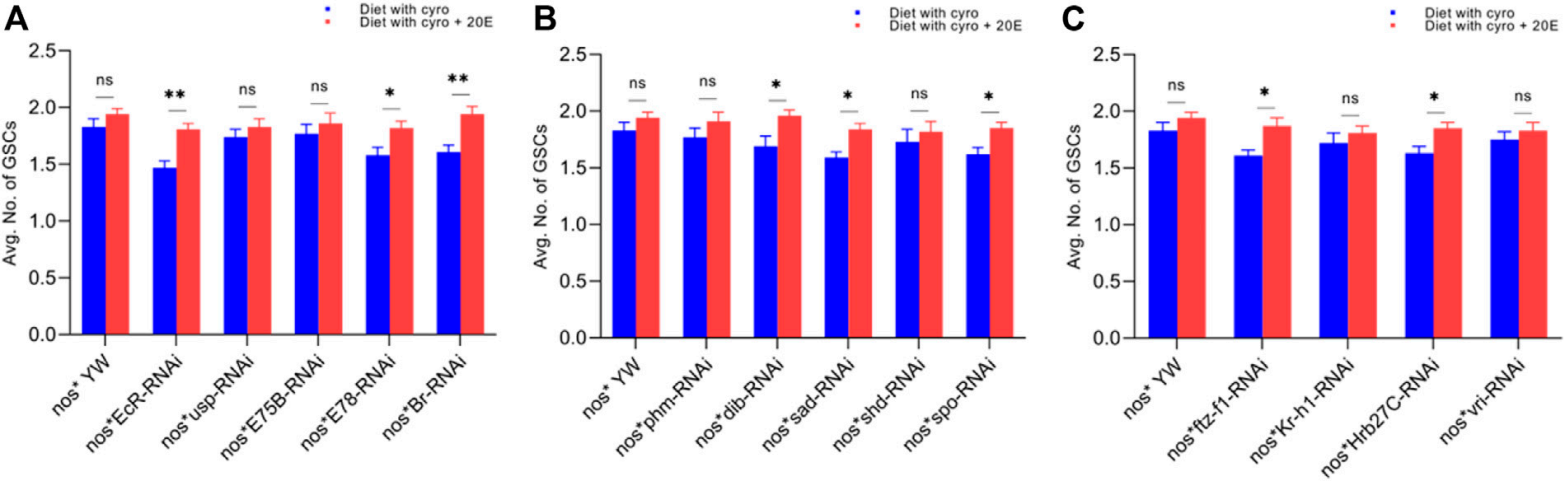
Rescue experiment of GSCs. **(A)** The number of GSCs against ecdysone-responsive primary genes. **(B)** The number of GSCs against Halloween genes. **(C)** The number of GSCs against ecdysone-responsive genes. The blue bars indicate the numbers of GSCs against the cyromazine treatment, while red bars indicate the numbers of GSCs from the flies fed on a diet containing 20E with the cyromazine. For the *t*-test: *p < 0.05; **p < 0.01; ***p < 0.001; ns indicates not significant (*p* > 0.05).

**FIGURE 7:**
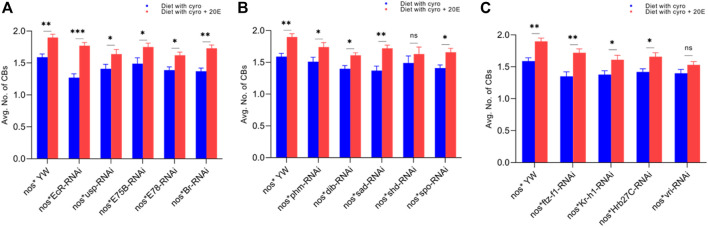
Rescue experiment of CBs. **(A)** The number of CBs against ecdysone-responsive primary genes. **(B)** The number of CBs against Halloween genes. **(C)** The number of CBs against ecdysone-responsive genes. The blue bars indicate the number of CBs against the cyromazine treatment, while the red bars indicate the number of CBs from the flies fed on a diet containing 20E with the cyromazine. For the *t*-test: *p < 0.05; **p < 0.01; ***p < 0.001; ns indicates not significant (*p* > 0.05).

To further support our experiment that the cyromazine decreased the germ cells in both the larval and adult ovaries by affecting the ecdysone signaling pathway, we performed rescue experiments by mixing exogenous 20E with a cyromazine-containing diet. We observed that the addition of 20E remarkably decreased the effect of cyromazine on the germ cells of larvae and adult ovaries (Figures 5–7).

We observed that the number of PGCs, against nos-Gal4* yw, increased from 79 ± 5 to 101 ± 7 after mixing 20E in the cyromazine-containing diet (Figure 5A). However, among treated transgenic lines, maximum numbers of PGCs were rescued against *Br* mutant flies (54.1%) (Figure 5A) followed by *sad* (51.8%) (Figure 5B) and *EcR* (51%) mutant flies (Figure 5A).

Furthermore, the maximum numbers of GSCs were rescued against *EcR* mutant flies (23%), followed by *Br* (20.5%) (Figure 6A) and *sad* (17.7%) (Figure 6B). The maximum numbers of CBs were also rescued against *EcR* (39.3%) (Figure 7A), followed by *ftz-f1* (27.4%) (Figure 7C) and *Br* (26.2%) (Figure 7A), after the addition of 20E.

These results indicated that the cyromazine significantly reduced the germ cell number in the larval and adult ovaries through interference in the ecdysone signaling pathway, as the addition of 20E notably rescued the germ cells. However, the addition of exogenous 20E in the control group showed no significant effect on the germ cells of *D. melanogaster* (results not shown).

### 3.7 TUNEL assay to detect apoptosis

We further performed the TUNEL assay to see if the reduction in germ cell number was associated with apoptosis. However, no positive apoptotic signal was observed in the ovaries of *D. melanogaster* treated with cyromazine, indicating that the decrease in the number of germ cells is not due to cell death (results not shown).

## 4 Discussion

Ecdysone signaling is crucial for insect reproduction (Kannangara et al., 2021). In *Drosophila*, both ecdysone receptor (*EcR*) and ultraspiracle (*usp*) control early response genes’ expression. These early genes later activate transcription factors which regulate the late-response genes to produce a tissue-specific response (Gauhar et al., 2009; Mazina et al., 2017). The ovary is the crucial source of ecdysone production in female adults (Lenaerts et al., 2019). Furthermore, ecdysone signaling is necessary for not only the proper ovary development but also for the maintenance and proliferation of GSCs (Ables and Drummond-Barbosa, 2010; Khalid et al., 2021). The present study also supported that ecdysone signaling is required to properly maintain germ cells in the *D. melanogaster*’s ovary. Mutations in *EcR*, *usp*, *E75B*, *E78*, and *Br* affected the GSCs’ maintenance and interfered with the germ cell differentiation and cyst development (König and Shcherbata, 2015; Ables et al., 2016). In addition, *E78* has been known to interact with *EcR*. The germ cell-specific knockdown of *E78* significantly reduced female fertility and also decreased the number of GSCs (Ables et al., 2015). Thus, the proper regulation of ecdysone signaling is essential for proper germ cell maintenance and proliferation.

In insects, cyromazine effects both metamorphosis and reproduction (Zhou et al., 2016). The mode of action of cyromazine has been reported to be related to the ecdysone signaling pathway. As in *Drosophila*’s larvae, the exogenous application of 20E significantly reduced the determinantal effect of cyromazine and also caused the early emergence of the adults (Van De Wouw et al., 2006). Previously, we observed that the continuous selection of cyromazine affected the reproduction of *Drosophila* by decreasing the number of GSCs and CBs in the ovary of a 3-day-old female. In the present study, we observed that the cyromazine also decreased the number of PGCs in the larvae ovaries, implying that the cyromazine affected the number of germ cells in the adult ovary by decreasing the numbers of PGCs in the larvae ovaries. In addition, RNA-Seq has been extensively utilized to detect changes in gene expression under various conditions, including insects treated with insecticides (Wei et al., 2019; Shu et al., 2021). Furthermore, no ovary-specific transcriptome study after treatment with cyromazine has been performed before. Therefore, to deeply understand how this insect growth regulator affected the germ cells of *D. melanogaster*, we performed tissue-specific next-generation RNA-Seq. Comparative transcriptomic analyses, between control and cyromazine-treated larval and adult ovaries of *D. melanogaster*, indicated a total of 26 and 863 DEGs, respectively. Furthermore, we observed that the ecdysone signaling related genes were differentially expressed after treatment with the cyromazine, indicating that the cyromazine significantly affected the ecdysone signaling pathway.

Later, we selected 14 key ecdysone-responsive genes and performed germ cell-specific RNA interference of selected genes to further validate our experiment. The results indicated a remarkable reduction in the number of germ cells among the cyromazine-treated transgenic lines as compared to nos-Gal4 * yw. We divided these selected genes into three categories: primary genes, Halloween genes, and ecdysone-responsive genes. Among the primary genes, significant numbers of PGCs, GSCs, and CBs were decreased against the cyromazine-treated *EcR* and E78 mutant flies (40.5%, 19.6%, and 20.1%, respectively), as compared to the nos-Gal4 * yw. However, no significant decrease in the numbers of PGCs, GSCs, and CBs was observed against *E75B* mutant flies compared to the nos-Gal4 * yw. Previous studies have reported that germ cells directly receive ecdysone signaling and inhibition of which significantly affect their maintenance. In addition, *E78* is required for the establishment of required numbers of GSCs (Ables et al., 2015). However, the mutation in *E75B* produced little effect on the maintenance of germ cells, indicating that *E75B* is not required in germ cells for their proper maintenance (Ables and Drummond-Barbosa, 2010). While among Halloween genes, the maximum number of PGCs, GSCs, and CBs were decreased against cyromazine-treated *sad* mutant flies, as compared to the nos-Gal4 * yw. However, no significant decrease in the number of PGCs, GSCs, and CBs was observed against *phm* and *shd* mutant flies. Such results can be supported by previous findings where the germ cells received the required amount of ecdysone signaling even in the absence of *phm* and *shd* (Domanitskaya et al., 2014). Furthermore, significant number of PGCs, GSCs, and CBs were reduced against *ftz-f1* and *Hrb27C* mutant flies. The mRNA expression of *Hrb27C* in the *EcR* mutant flies indicated that it is ecdysone-responsive. In addition, more than 60% loss in the GSCs was observed in *Hrb27C* mutant flies (Ables et al., 2016). Likewise, *ftz-f1* has also been reported to be ecdysone-responsive, the loss of which affects the number of germ cells (Sanders, 2022), while no significant decrease in the numbers of PGCs and GSCs was observed against *Kr-h1* mutant flies, which further supported that the decrease in the numbers of germ cells is due to the ecdysone signaling pathway. *Kr-h1* is a juvenile hormone (JH) response transcriptional factor which inhibits *Br* expression to decrease ecdysone signaling (Jiang et al., 2017; He et al., 2020).

In *D. melanogaster*, ecdysone signaling is necessary to suppress the PGC differentiation (Gancz et al., 2011). In addition, the mutations in primary response genes also decreased oogenesis (Belles and Piulachs, 2015). Furthermore, RNAi-mediated mutations in the other ecdysone-responsive genes such as *Hrb27C*, *vkg*, *Acer*, *Trn-SR*, *CG12050*, *MESR3*, and *CycE* resulted in more than 50% loss in the GSC numbers, as compared to the control (Ables et al., 2016). Furthermore, it has been reported that the maintenance of CBs, formation, and encapsulation of cysts are also controlled by ecdysone (König et al., 2011; Morris and Spradling, 2012; Xuan et al., 2013; Ables et al., 2015; König and Shcherbata, 2015). Likewise, further decrease in the number of germ cells against the cyromazine-treated transgenic lines further supported that the effect of the cyromazine on the germ cells is through the ecdysone signaling pathway.

The ecdysone hormone is known to control the stem cell fate (intestinal stem cells) in the *Drosophila*’s ovary (Zipper et al., 2020). In addition, we observed that the feeding of exogenous 20E significantly reduced the chemical’s effect on the germ cells of both the larval and adult ovaries (Figures 5–7). Among the primary response genes, number of GSCs were significantly rescued against the *EcR*, *E78*, and *Br* mutant flies fed on a diet containing 20E, as compared to flies fed on a diet containing cyromazine alone, similarly significant numbers of PGCs and CBs were rescued against nos-Gal4 * yw and *usp* mutant flies. However, no significant difference in the numbers of GSCs was observed against nos-Gal4 * yw and *usp* mutant flies fed on a diet containing exogenous 20E (Figure 4A). A significant number of PGCs, GSCs, and CBs were rescued against *sad* and *spo* mutant flies fed on a diet containing 20E, as compared to flies fed on a diet containing cyromazine alone. No significant increase in the number of PGCs and GSCs was observed against the *shd* mutant flies (Figures 5A,B). However, a significant number of PGCs were rescued against all selected primary genes of ecdysone signaling (Figure 4). Likewise, a significant number of CBs were rescued against selected ecdysone-responsive genes except for *vri* mutant flies (Figure 7C). However, a significant increase in the number of GSCs was only observed against the *ftz-f1* and *Hrb27C* mutant flies fed on a diet containing 20E (Figure 6C). Such results were observed previously, where the exogenous application of 20E rescued the GSCs due to mutation in *Hrb27C* (Ables et al., 2016).

The exogenous application of 20E not only decreased the larval mortality but also caused early eclosion of adult flies (Van De Wouw et al., 2006). Thus, our results supported that the effect of cyromazine, on the germ cells of *D. melanogaster*, is through the ecdysone signaling pathway.

## 5 Conclusion

In this study, the ovarian transcriptome of *D. melanogaster* was systematically analyzed at both larval and adult stages, after treatment with cyromazine. Data analysis showed that the ecdysone signaling pathway was significantly affected due to cyromazine stress. Subsequently, we selected 14 genes that were receptive to ecdysone signaling, and solely inhibited their expression in the germ cells. Results revealed a significant decrease in the number of germ cells against the cyromazine fed transgenic lines, as compared to the cyromazine fed nos-Gal4 * yw. To restore ecdysone signaling, we mixed exogenous 20E with the cyromazine-containing diet. Our findings confirmed that the administration of exogenous 20E significantly rescued the germ cells, thereby suggesting that the cyromazine affected the germ cells by the ecdysone signaling pathway.

## Data Availability

The data presented in the study are deposited in the NCBI repository, accession number PRJNA865070.

## References

[B1] Ables E. T. Bois K. E. Garcia C. A. Drummond-Barbosa D. J. D. B. (2015). Ecdysone response gene *E78* controls ovarian germline stem cell niche formation and follicle survival in *Drosophila* . Dev. Biol. 400, 33–42. 10.1016/j.ydbio.2015.01.013 25624267 PMC4448935

[B2] Ables E. T. Drummond-Barbosa D. J. C. S. C. (2010). The steroid hormone ecdysone functions with intrinsic chromatin remodeling factors to control female germline stem cells in *Drosophila* . Cell stem Cell 7, 581–592. 10.1016/j.stem.2010.10.001 21040900 PMC3292427

[B3] Ables E. T. Hwang G. H. Finger D. S. Hinnant T. D. Drummond-Barbosa D. J. G. G. (2016). A genetic mosaic screen reveals ecdysone-responsive genes regulating *Drosophila* oogenesis. G3 6, 2629–2642. 10.1534/g3.116.028951 27226164 PMC4978916

[B4] Belles X. Piulachs M. D. J. B. E. B. a.-G. R. M. (2015). Ecdysone signalling and ovarian development in insects: from stem cells to ovarian follicle formation. Biochim. Biophys. Acta 1849, 181–186. 10.1016/j.bbagrm.2014.05.025 24939835

[B5] Ben-Zvi D. S. Volk T. J. B. O. (2019). Escort cell encapsulation of *Drosophila* germline cells is maintained by irre cell recognition module proteins. Biol. Open 8, bio039842. 10.1242/bio.039842 30837217 PMC6451344

[B6] Cao X. Jiang H. J. B. G. (2017). An analysis of 67 RNA-seq datasets from various tissues at different stages of a model insect, *Manduca sexta* . BMC Genomics 18, 796–814. 10.1186/s12864-017-4147-y 29041902 PMC5645894

[B7] Domanitskaya E. Anllo L. Schüpbach T. (2014). Phantom, a cytochrome P450 enzyme essential for ecdysone biosynthesis, plays a critical role in the control of border cell migration in *Drosophila* . Dev. Biol. 386 (2), 408–418. 10.1016/j.ydbio.2013.12.013 24373956 PMC4028970

[B8] Drummond-Barbosa D. J. G. (2019). Local and physiological control of germline stem cell lineages in *Drosophila melanogaster* . Genetics 213, 9–26. 10.1534/genetics.119.300234 31488592 PMC6727809

[B9] Eliazer S. Buszczak M. (2011). Finding a niche: studies from the *Drosophila* ovary. Stem Cell Res. Ther. 2, 45–48. 10.1186/scrt86 22117545 PMC3340554

[B10] Eliazer S. Palacios V. Wang Z. Kollipara R. K. Kittler R. Buszczak M. J. P. G. (2014). *Lsd1* restricts the number of germline stem cells by regulating multiple targets in escort cells. PLoS Genet. 10, e1004200. 10.1371/journal.pgen.1004200 24625679 PMC3952827

[B11] Gancz D. Lengil T. Gilboa L. J. P. B. (2011). Coordinated regulation of niche and stem cell precursors by hormonal signaling. PLoS Biol. 9, e1001202. 10.1371/journal.pbio.1001202 22131903 PMC3222635

[B12] Gao Y. Mao Y. Xu R. G. Zhu R. Zhang M. Sun J. (2019). Defining gene networks controlling the maintenance and function of the differentiation niche by an *in vivo* systematic RNAi screen. J. Genet. Genomics. 46, 19–30. 10.1016/j.jgg.2018.10.008 30745214

[B13] Gauhar Z. Sun L. V. Hua S. Mason C. E. Fuchs F. Li T.-R. (2009). Genomic mapping of binding regions for the Ecdysone receptor protein complex. Genome Res. 19, 1006–1013. 10.1101/gr.081349.108 19237466 PMC2694480

[B14] Gilboa L. Lehmann R. J. C. B. (2004). Repression of primordial germ cell differentiation parallels germ line stem cell maintenance. Curr. Biol. 14, 981–986. 10.1016/j.cub.2004.05.049 15182671

[B15] Gilboa L. Lehmann R. J. N. (2006). Soma–germline interactions coordinate homeostasis and growth in the *Drosophila* gonad. Nature 443, 97–100. 10.1038/nature05068 16936717

[B16] He Q. Zhang Y. Dong W. (2020). MicroRNA miR‐927 targets the juvenile hormone primary response gene Krüppel homolog1 to control *Drosophila* developmental growth. Insect Mol. Biol. 29, 545–554. 10.1111/imb.12662 32715555

[B17] Hinnant T. D. Merkle J. A. Ables E. T. J. F. I. C. Biology D. (2020). Coordinating proliferation, polarity, and cell fate in the *drosophila* female germline. Front. Cell Dev. Biol. 8, 19. 10.3389/fcell.2020.00019 32117961 PMC7010594

[B18] Huang J. Reilein A. Kalderon D. J. D. (2017). Yorkie and Hedgehog independently restrict BMP production in escort cells to permit germline differentiation in the *Drosophila* ovary. Development 144, 2584–2594. 10.1242/dev.147702 28619819 PMC5536926

[B19] Ji S. Li C. Hu L. Liu K. Mei J. Luo Y. (2017). Bam-dependent deubiquitinase complex can disrupt germ-line stem cell maintenance by targeting cyclin A. Proc. Natl. Acad. Sci. U. S. A. 114, 6316–6321. 10.1073/pnas.1619188114 28484036 PMC5474830

[B20] Jiang J. Xu Y. Lin X. (2017). Role of Broad-Complex (Br) and Krüppel homolog 1 (Kr-h1) in the ovary development of Nilaparvata lugens. Front. Physiol. 8, 1013. 10.3389/fphys.2017.01013 29270133 PMC5724046

[B21] Jin Z. Kirilly D. Weng C. Kawase E. Song X. Smith S. (2008). Differentiation-defective stem cells outcompete normal stem cells for niche occupancy in the *Drosophila* ovary. Cell Stem Cell 2, 39–49. 10.1016/j.stem.2007.10.021 18371420 PMC8387725

[B22] Kannangara J. R. Mirth C. K. Warr C. G. J. O. B. (2021). Regulation of ecdysone production in *Drosophila* by neuropeptides and peptide hormones. Open Biol. 11, 200373. 10.1098/rsob.200373 33593157 PMC8103234

[B23] Khalid M. Z. Ahmad S. Ngegba P. M. Zhong G. J. B. (2021). Role of endocrine system in the regulation of female insect reproduction. Biology 10, 614. 10.3390/biology10070614 34356469 PMC8301000

[B24] Khalid M. Z. Sun Z. Chen Y. Zhang J. Zhong G. (2022). Cyromazine effects the reproduction of *Drosophila* by decreasing the number of germ cells in the female adult ovary. Insects 13 (5), 414. 10.3390/insects13050414 35621750 PMC9144682

[B25] Khan H. a. A. Akram W. J. C. (2017). Cyromazine resistance in a field strain of house flies, *Musca domestica L.*: Resistance risk assessment and bio-chemical mechanism. Chemosphere 67, 308–313. 10.1016/j.chemosphere.2016.10.018 27728890

[B26] Kim D. Langmead B. Salzberg S. L. J. N. M. (2015). HISAT: a fast spliced aligner with low memory requirements. Nat. Methods 12, 357–360. 10.1038/nmeth.3317 25751142 PMC4655817

[B27] König A. Shcherbata H. R. J. B. O. (2015). Soma influences GSC progeny differentiation via the cell adhesion-mediated steroid-let-7-Wingless signaling cascade that regulates chromatin dynamics. Biol. Open 4, 285–300. 10.1242/bio.201410553 25661868 PMC4359735

[B28] König A. Yatsenko A. S. Weiss M. Shcherbata H. R. J. T. E. J. (2011). Ecdysteroids affect *Drosophila* ovarian stem cell niche formation and early germline differentiation. EMBO J. 30, 1549–1562. 10.1038/emboj.2011.73 21423150 PMC3102283

[B29] Lenaerts C. Marchal E. Peeters P. Broeck J. V. J. S. R. (2019). The ecdysone receptor complex is essential for the reproductive success in the female desert locust, *Schistocerca gregaria* . Sci. Rep. 9, 15–12. 10.1038/s41598-018-36763-9 30626886 PMC6327042

[B30] Li M. Hu X. Zhang S. Ho M. S. Wu G. Zhang L. J. S. R. (2019). Traffic jam regulates the function of the ovarian germline stem cell progeny differentiation niche during pre-adult stage in Drosophila. Sci. Rep. 9, 10124–10216. 10.1038/s41598-019-45317-6 31300663 PMC6626045

[B31] Liu Z. Zhong G. Chai P. C. Luo L. Liu S. Yang Y. (2015). Coordinated niche-associated signals promote germline homeostasis in the *Drosophila* ovary. J. Cell Biol. 211, 469–484. 10.1083/jcb.201503033 26504174 PMC4621830

[B32] Mao Y. Tu R. Huang Y. Mao D. Yang Z. Lau P. K. (2019). The exocyst functions in niche cells to promote germline stem cell differentiation by directly controlling EGFR membrane trafficking. Development 146, dev174615. 10.1242/dev.174615 31142545 PMC6633608

[B33] Mazina M. Y. Kocheryzhkina E. Nikolenko J. Krasnov A. Georgieva S. Vorobyeva N. (2017). Nuclear receptors *EcR, Usp, E75, DHR3*, and *ERR* regulate transcription of ecdysone cascade genes. Dokl. Biochem. Biophys. 473, 145–147. 10.1134/S1607672917020144 28510140

[B34] Morris L. X. Spradling A. C. (2012). Steroid signaling within *Drosophila* ovarian epithelial cells sex-specifically modulates early germ cell development and meiotic entry. PLoS ONE 7, e46109. 10.1371/journal.pone.0046109 23056242 PMC3462805

[B35] Panchal T. Chen X. Alchits E. Oh Y. Poon J. Kouptsova J. (2017). Specification and spatial arrangement of cells in the germline stem cell niche of the *Drosophila* ovary depend on the Maf transcription factor Traffic jam. PLoS Genet. 13, e1006790. 10.1371/journal.pgen.1006790 28542174 PMC5459507

[B36] Sanders S. O. (2022). “The role of Drosophila nuclear receptor ftz-f1 in somatic cells is neccessary for germline stem cell maintenance,” [Master's Thesis]. (Greenville (NC): East Carolina University).

[B37] Sato T. Ogata J. Niki Y. J. Z. S. (2010). BMP and Hh signaling affects primordial germ cell division in *Drosophila* . Zool. Sci. 27, 804–810. 10.2108/zsj.27.804 20887178

[B38] Shu B. Yu H. Li Y. Zhong H. Li X. Cao L. (2021). Identification of azadirachtin responsive genes in Spodoptera frugiperda larvae based on RNA-seq. Pestic. Biochem. Physiol. 172, 104745. 10.1016/j.pestbp.2020.104745 33518039

[B39] Taylor D. B. Friesen K. Zhu J. J. Sievert K. J. J. O. E. E. (2012). Efficacy of cyromazine to control immature stable flies (Diptera: Muscidae) developing in winter hay feeding sites. J. Econ. Entomol. 105, 726–731. 10.1603/ec11317 22606846

[B40] Ting X. J. W. I. R. D. B. (2013). Control of germline stem cell self‐renewal and differentiation in the *Drosophila* ovary: concerted actions of niche signals and intrinsic factors. Wiley Interdiscip. Rev. Dev. Biol. 2, 261–273. 10.1002/wdev.60 24009036

[B41] Van De Wouw A. P. Batterham P. Daborn P. (2006). The insect growth regulator insecticide cyromazine causes earlier emergence in *Drosophila melanogaster* . Arch. Insect Biochem. Physiol. 63, 101–109. 10.1002/arch.20146 17048245

[B42] Weaver L. N. Drummond-Barbosa D. J. G. (2018). Maintenance of proper germline stem cell number requires adipocyte collagen in adult *Drosophila* females. Genetics 209, 1155–1166. 10.1534/genetics.118.301137 29884747 PMC6063239

[B43] Wei N. Zhong Y. Lin L. Xie M. Zhang G. Su W. (2019). Transcriptome analysis and identification of insecticide tolerance-related genes after exposure to insecticide in *Sitobion avenae* . Genes 10 (12), 951. 10.3390/genes10120951 PMC6947367 31757092

[B44] Xuan T. Xin T. He J. Tan J. Gao Y. Feng S. (2013). dBre1/dSet1-dependent pathway for histone H3K4 trimethylation has essential roles in controlling germline stem cell maintenance and germ cell differentiation in the *Drosophila* ovary. Dev. Biol. 379, 167–181. 10.1016/j.ydbio.2013.04.015 23624310

[B45] Yatsenko A. S. Shcherbata H. R. J. P. G. (2021). Distant activation of Notch signaling induces stem cell niche assembly. PLoS Genet. 17, e1009489. 10.1371/journal.pgen.1009489 33780456 PMC8031783

[B46] Yoshinari Y. Ameku T. Kondo S. Tanimoto H. Kuraishi T. Shimada-Niwa Y. (2020). Neuronal octopamine signaling regulates mating-induced germline stem cell increase in female *Drosophila melanogaster* . Elife 9, e57101. 10.7554/eLife.57101 33077027 PMC7591258

[B47] Zafar J. Zhang Y. Huang J. Freed S. Shoukat R. F. Xu X. (2021). Spatio-temporal profiling of *Metarhizium anisopliae*—responsive microRNAs involved in modulation of plutella xylostella immunity and development. J. Fungi 7, 942. 10.3390/jof7110942 PMC8620415 34829229

[B48] Zhou F. Zhu G. Zhao H. Wang Z. Xue M. Li X. (2016). Sterilization effects of adult-targeted baits containing insect growth regulators on *Delia antiqua* . Sci. Rep. 6, 32855–32859. 10.1038/srep32855 27619006 PMC5020654

[B49] Zhu C.-H. Xie T. (2003). Clonal expansion of ovarian germline stem cells during niche formation in Drosophila. Development 130 (12), 2579–2588. 10.1242/dev.00499 12736203

[B50] Zipper L. Jassmann D. Burgmer S. Görlich B. Reiff T. J. E. (2020). Ecdysone steroid hormone remote controls intestinal stem cell fate decisions via the PPARγ-homolog Eip75B in *Drosophila* . Elife 9, e55795. 10.7554/eLife.55795 32773037 PMC7440922

