# Supplementary figures and images for: Cyromazine affects the ovarian germ cells of Drosophila via the ecdysone signaling pathway
Source: Front Physiol. 2022 Sep 29;13:992306. doi: 10.3389/fphys.2022.992306 (PMC9557234; doi:10.3389/fphys.2022.992306)

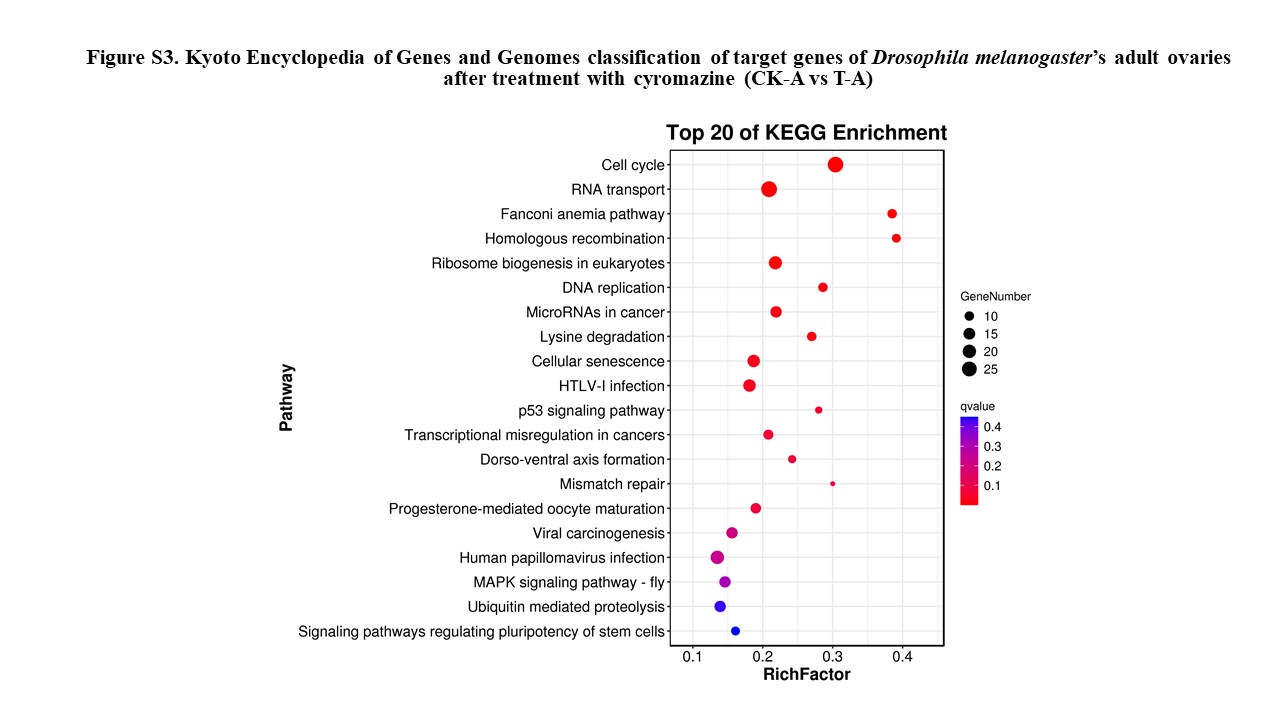

Supplement: Supplementary file 2 [file Image3.jpg]

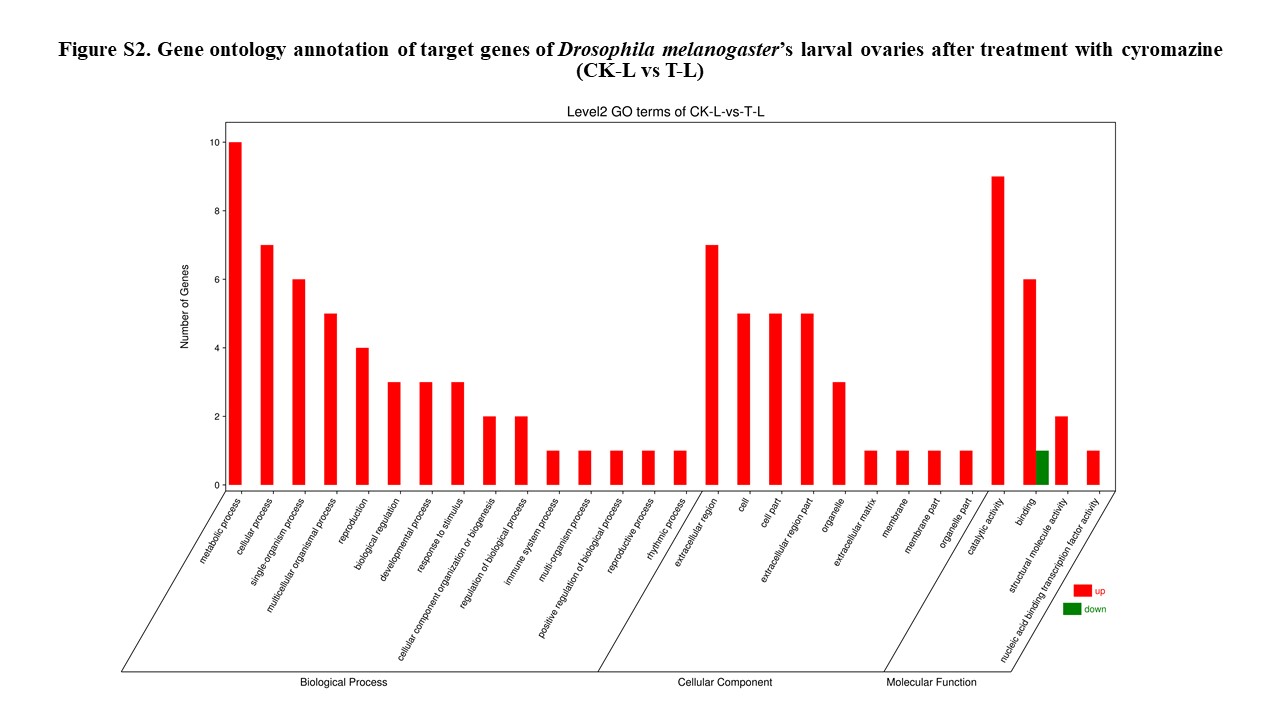

Supplement: Supplementary file 3 [file Image2.jpg]

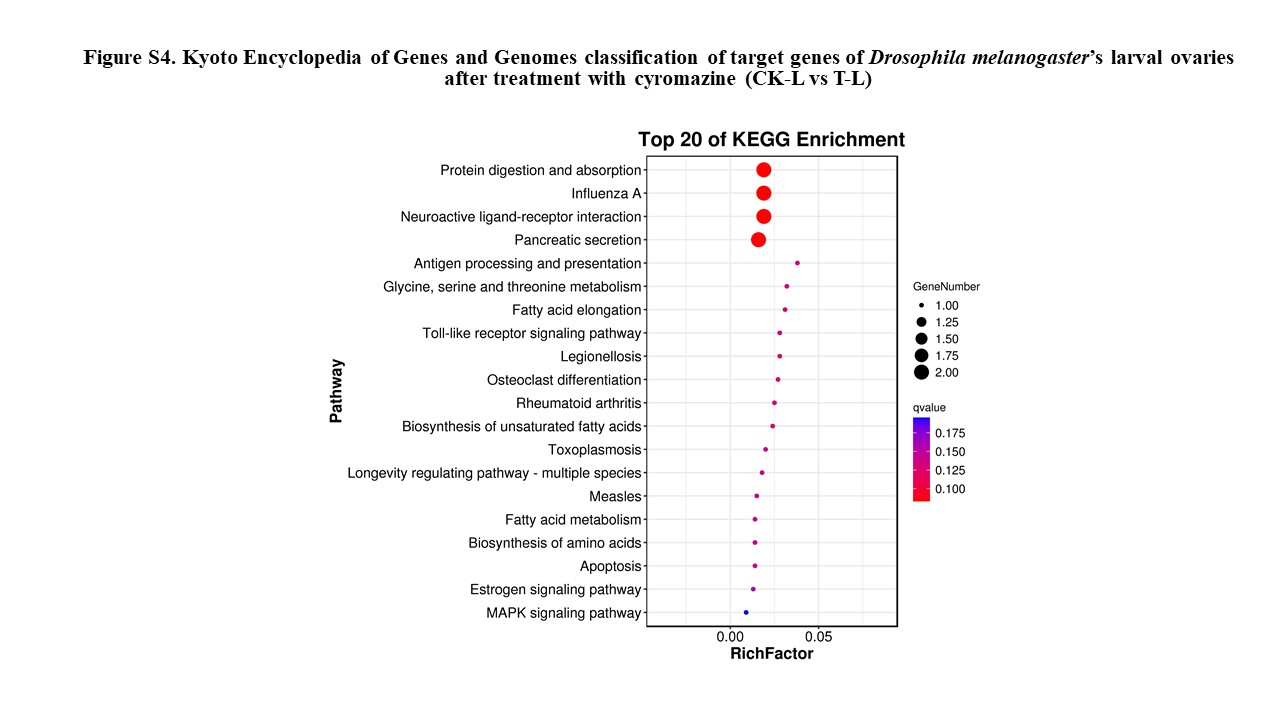

Supplement: Supplementary file 7 [file Image4.jpg]

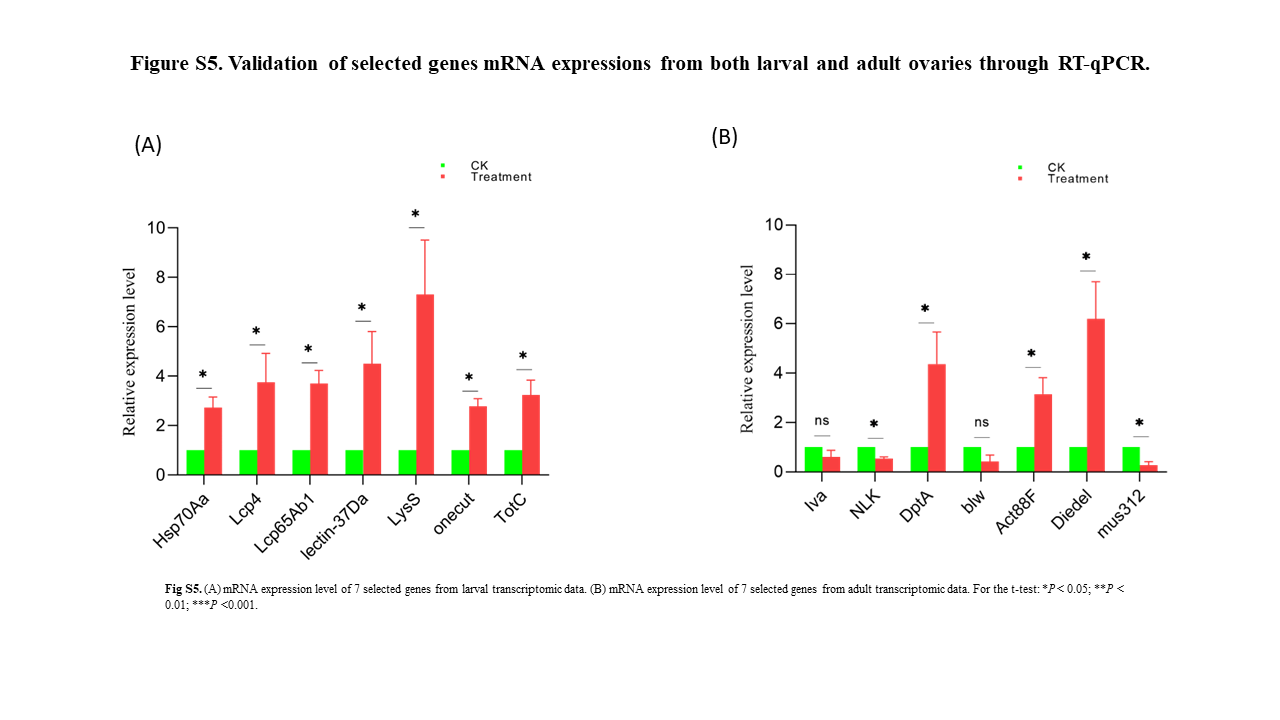

Supplement: Supplementary file 8 [file Image5.tif]

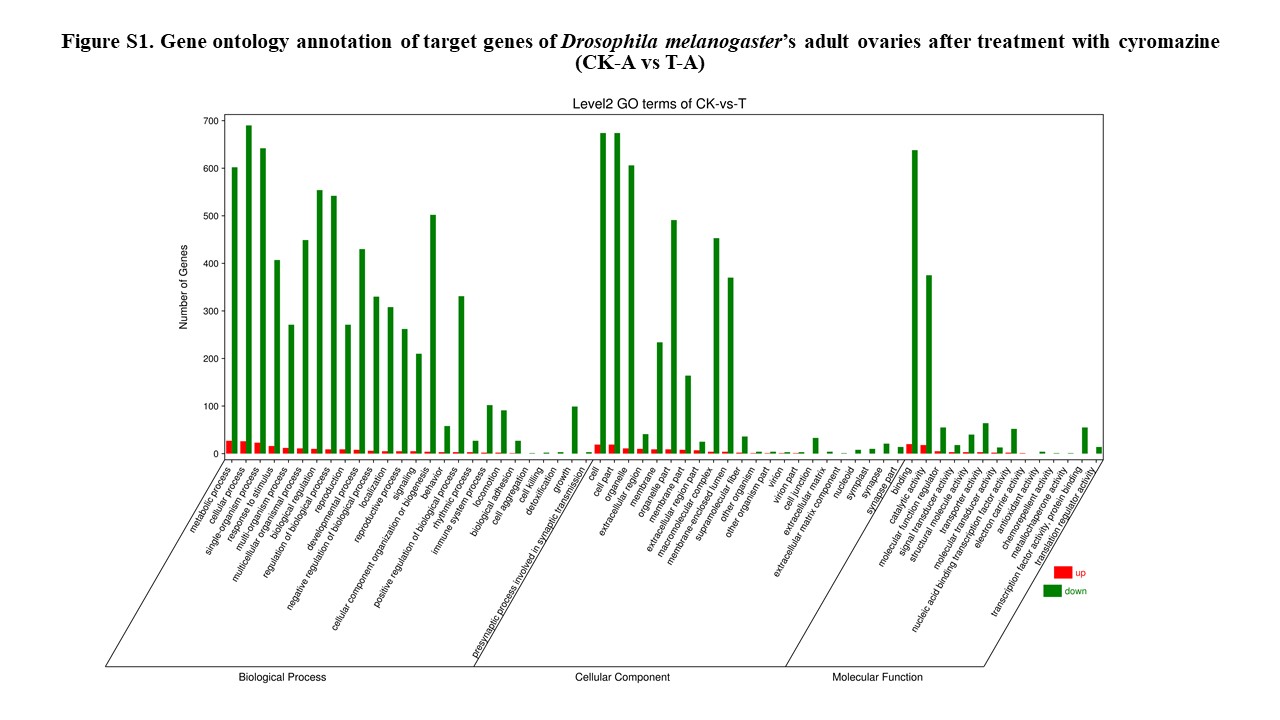

Supplement: Supplementary file 10 [file Image1.jpg]
