# Supplementary material for: Cyromazine affects the ovarian germ cells of Drosophila via the ecdysone signaling pathway
Source: Front Physiol. 2022 Sep 29;13:992306. doi: 10.3389/fphys.2022.992306 (PMC9557234; doi:10.3389/fphys.2022.992306)
Supplement: Supplementary file 4 [file Table1.docx]

Supplementary Table S1. Flies stock.

UAS-EcR-RNAi (V37058), UAS-usp-RNAi (BS27258), UAS-Br-RNAi (BS27272), UAS-E75B-RNAi (BS26717) and UAS-E78-RNAi (BS35780) were provided by Dr. Suning Liu (South China Normal University, China), while yw and nos-Gal4 were previously maintained in our laboratory (Khalid et al. 2022).

The following transgenic lines were purchased from TsingHua Fly Center.

| Serial No. | TH number | Annotation Symbol | Gene Symbol |
| --- | --- | --- | --- |
| 01. | TH05011.N | CG12028 | dib |
| 02. | TH05022.N | CG13478 | shd |
| 03. | THU3583 | CG10377 | Hrb27C |
| 04. | THU5252 | CG14029 | vri |
| 05. | TH04817.N | CG6578 | phm |
| 06. | TH01976.N | CG18783 | Kr-h1 |
| 07. | TH05013.N | CG14728 | Sad |
| 08. | TH01848.N | CG10594 | spo |
| 09. | THU0560 | CG4059 | ftz-f1 |
