# Supplementary material for: Cyromazine affects the ovarian germ cells of Drosophila via the ecdysone signaling pathway
Source: Front Physiol. 2022 Sep 29;13:992306. doi: 10.3389/fphys.2022.992306 (PMC9557234; doi:10.3389/fphys.2022.992306)
Supplement: Supplementary file 5 [file Table2.docx]

| Name | Primer Sequences (*5’*-*3*’) |
| --- | --- |
| Lcp4 | gaacacgtccgtgtgagcta |
|  | agggcaaggtctttggagtt |
| TotC | ttgccctgctcctgattagt |
|  | gcgcctaaagatatcgagca |
| onecut | caagaacccagttccgttgt |
|  | actgtcccttttggtgatgc |
| lectin-37Da | cacggagtccttgaactggt |
|  | gcattggtgaaccatctgtg |
| LysS | ttctttgctctggtgctcct |
|  | ccaggtgcggtagtcactct |
| Hsp70Aa | cattccgtgcaagcagacta |
|  | gctgacgttcaggattccat |
| Lcp65Ab1 | acaaacaccacacagctcca |
|  | gaggacaccctcctgtttga |
| Iva | ctaaagccgccaagcttatg |
|  | ttcctgctgtcagaacatcg |
| blw | ttgcagcacatcaagacctc |
|  | ttcttcgtttgctgctgatg |
| mus312 | ccaaatgacaccgaacacag |
|  | agagcagaattgcgtcgttt |
| NLK | caccgcatttggatttcttt |
|  | tcgagttgaccagcagattg |
| Act88F | atccgcaaggatctgtatgc |
|  | ttcgagatccacatctgctg |
| Diedel | tatggcgagtcatttgtgga |
|  | cttcgaatcgctctggtagg |
| DptA | accgcagtacccactcaatc |
|  | actttccagctcggttctga |

Supplementary Table S2: Primer’s sequence
